# Paternal High-Fat Diet Altered Sperm 5'tsRNA-Gly-GCC Is Associated With Enhanced Gluconeogenesis in the Offspring

**DOI:** 10.3389/fmolb.2022.857875

**Published:** 2022-04-11

**Authors:** Bin Wang, Lin Xia, Dan Zhu, Hongtao Zeng, Bin Wei, Likui Lu, Weisheng Li, Yajun Shi, Jingliu Liu, Yunfang Zhang, Miao Sun

**Affiliations:** ^1^ Institute for Fetology, The First Affiliated Hospital of Soochow University, Suzhou City, China; ^2^ Medical Center of Hematology, The Xinqiao Hospital of Army Medical University, Chongqing, China; ^3^ Shanghai Key Laboratory of Signaling and Disease Research, School of Life Sciences and Technology, Clinical and Translational Research Center of Shanghai First Maternity and Infant Hospital, Tongji University, Shanghai, China

**Keywords:** paternal high-fat diet, 5′tsRNA-Gly-GCC, gluconeogenesis, Sirt6, FoxO1

## Abstract

**Background:** Paternal lifestyle, stress and environmental exposures play a crucial role in the health of offspring and are associated with non-genetic inheritance of acquired traits, however the underlying mechanisms are unclear. In this study, we aimed to find out how the sperm tsRNA involved in paternal high-fat diet induced abnormal gluconeogenesis of F1 offspring, and explore the underlying molecular mechanism of its regulation.

**Method:** We generated a paternal high fat diet (42% kcal fat) model to investigate the mechanism by which paternal diet affects offspring metabolism. Four-week-old C57BL/6J male mice were randomly assigned into two groups to receive either a control diet (CD; 10% kcal fat) or a high-fat (HFD; 42% kcal fat) diet for 10 weeks, and mice from each group were then mated with 8-week-old females with control diet in a 1:2 ratio to generate F1. F0 sperms were isolated and small RNAs was sequenced by high-throughput sequencing. Metabolic phenotypes were examined with both F0 and F1.

**Results:** A significant increase in body weight was observed with HFD-F0 mice at 8 weeks of age as compared to CD mice at the same age. F0 mice showed impaired glucose tolerance (GTT), resistance to insulin tolerance (ITT) and increased pyruvate tolerance (PTT) at 14 weeks. HFD-F1 male mice showed no significant difference in body weight. An increase in PTT was found at 13 weeks of age and no significant changes in GTT and ITT. PEPCK and G6Pase that related to gluconeogenesis increased significantly in the liver of HFD-F1 male mice. Sperm sequencing results showed that 5′tsRNA-Gly-GCC derived from tRNA-Gly-GCC-2 specifically was remarkably upregulated in sperm of HFD F0 mice. Q-PCR further showed that this tsRNA was also increased in the liver of HFD-F1 comparison with CD-F1 mice. In addition, we found that 5′tsRNA-Gly-GCC can regulate Sirt6-FoxO1 pathway and be involved in the gluconeogenesis pathway in liver.

**Conclusion:** 5′tsRNA-Gly-GCC that increased in HFD mice mature sperms can promote gluconeogenesis in liver by regulating Sirt6-FoxO1 pathway, which might represent a potential paternal epigenetic factor mediating the intergenerational inheritance of diet-induced metabolic alteration.

## Introduction

Developmental origins of health and disease (DoHaD), originally proposed by David Barker in 1980s, has proved that many adult diseases, including hypertension, diabetes, obesity, cancer and other diseases, may be related to dysfunctions caused by intrauterine insult (Barker and Osmond, 1986; Li et al., 2017). The global prevalence of obesity has increased rapidly in the past four decades, among them, age-standardized prevalence of obesity increased from 3.2% in 1975 to 10.8% in 2014 in men, and from 6.4 to 14.9% in women. If these trends continue, by 2025, the global prevalence of male obesity will reach 18%, of which the severe obesity will surpass 6% (NCD Risk Factor Collaboration, 2016). Various epidemiological and experimental studies have confirmed the impact of maternal obesity on the health of offspring, including obesity, metabolic dysfunction, cardiovascular disease and behavioral alterations (Dunn and Bale, 2009; Gaillard et al., 2014; Gaillard, 2015; Contu and Hawkes, 2017; Kislal et al., 2020). In contrast, few studies have focused on the effects of father’s lifestyle and paternal environmental exposures on the health of offspring. Until recently, studies have found that paternal obesity plays an important role in the health of offspring. In human studies, paternal body mass index (BMI) is closely related to the growth and metabolic phenotype of offspring (Kaati et al., 2002; Chen et al., 2012); and animal studies have also found that paternal obesity can lead to offspring pancreatic islet dysfunction, growth deficit, dysregulated gluconeogenesis, diminished reproductive and gamete functions (Ng et al., 2010; Fullston et al., 2012; Lecomte et al., 2017; Wu et al., 2021). However, the underlying mechanism remains unclear. Liver plays an important role in maintaining systemic glucose homeostasis by regulating gluconeogenesis, glycogenolysis, glycogen synthesis, glycolysis and other pathways. Hepatic glucose production (HGP) accounts for about 90% of endogenous glucose production (Ekberg et al., 1999; Petersen et al., 2017). Therefore, it is necessary to explore the effect of paternal high-fat diet on hepatic gluconeogenesis in offspring.

A growing number of studies have shown that small non-coding RNAs (sncRNAs) are important regulators of various biological processes, such as cell differentiation and cellular responses to stress (Cai et al., 2013; Wang et al., 2020a). sncRNAs have a wide and far-reaching impact on biological systems. Among them, miRNA is the most studied sncRNAs subtype and plays a significant role in regulating gene expression (Bernstein et al., 2001). Because sncRNAs can silence gene expression in a specific and targeted manner, they have also been used as biological research and/or therapeutic intervention tools (Galasso et al., 2010). The improved sequencing platform and methods have recently made us realize that mature tRNAs can also produce sncRNA molecules *via* site specific cleavage by various RNase, such as angiogenin (ANG) and Dicer (Sun et al., 2018). These tRNA-derived small RNAs (tsRNAs), also known as tRNA fragments (tRFs), could participate in translation inhibition and play a regulatory role in a variety of physiological and pathological phenomena (Sun et al., 2018), and have also attracted progressively more attention in epigenetic research field recently (Gebetsberger and Polacek, 2013; Keam and Hutvagner, 2015; Wang et al., 2020b; Rashad et al., 2020).

More and more studies have found that tsRNAs play an important role in metabolic disorders (Chen et al., 2016; Wang et al., 2020a; Wang et al., 2020b). Mouse mature sperm contains a large amount of tsRNA, which is much higher than miRNA and piRNA (Peng et al., 2012). We previously found that the expression profile of tsRNAs in mature sperm have dynamically changed in the paternal mouse model given high-fat diet (HFD, 60% fat). Injection of sperm tsRNA fractions from HFD mice into normal zygotes led to metabolic disorder in F1 offspring and changed the gene expression of metabolic pathway in early embryos and islets of F1 offspring, which is independent of DNA methylation in CpG enrichment region (Chen et al., 2016). Moreover, in the maternal HFD model, sperm tsRNAs were increased in their F1 male offspring. Microinjection of sperm tsRNAs isolated from F1-HFD male mice into normal zygotes reproduced addictive-like behaviors and obesogenic phenotypes in the resultant offspring (Sarker et al., 2019). These results suggest that tsRNAs in sperm are carriers of epigenetic information that can transmit environmental exposure induced parental acquired phenotype to the next generation. In addition, as tRNA derived small non coding RNAs, sperm tsRNAs (30–40 nt) maintain various RNA modifications that inherited from mature tRNA, such as m^5^C, m^2^G, m^1^A, Ψ and others, which might contribute to tsRNA stability in zygotes (Chen et al., 2016). The deletion of mouse tRNA methyltransferase (DNMT2) prevented the increase of m^5^C and m^2^G levels in sperm 30–40 nt RNA fractions and the alteration of tsRNA profiles that are induced by HFD, which further abolished sperm tsRNA-mediated transmission of HFD-induced metabolic disorders to offspring (Zhang et al., 2018a). However, the mechanism of how sperm tsRNAs transmit the paternal metabolic alteration to the offspring is still under investigation.

In this study, we used high-throughput small RNA sequencing to identify differentially expressed tsRNA in sperm under high-fat diet (42% fat), and explore the mechanism of how tsRNAs contribute to the abnormal metabolic phenotypes in F1 offspring. Our data showed that 5′tsRNA-Gly-GCC (30 nt), which both increased in HFD F0 sperm and HFD F1 liver, could promote gluconeogenesis in F1 liver by regulating Sirt6-FoxO1 pathway.

## Methods and Materials

### Animals

C57BL/6J mice were maintained under specific-pathogen-free conditions at the experimental animal center of Soochow University. All mice were placed in cages with a maximum of five in a temperature-controlled room (22 ± 2°C) with a 12 h light/dark cycle and free access to food and water. Two groups of 4-week-old male mice were randomly assigned to receive a control diet (CD; 10% kcal fat) or a high-fat diet (HFD; 42% kcal fat). Both groups of mice were fed for 10 weeks and their body weight was monitored weekly, followed by metabolic test or offspring breeding. The males in each group mated with 8-week-old normal females in a ratio of 1:2 to produce F1 offspring. All procedures and protocols in this study were approved by the Institutional Animal Care and Use Committee of Soochow University.

### Metabolic Testing

#### Intraperitoneal Glucose Tolerance Test (IPGTT)

Mice were fasted overnight (16 h) before IPGTT. On the day of the experiment, the animals received intraperitoneal injection of 2 g glucose/kg body weight (de Castro Barbosa et al., 2016a). Blood collected from the tail was used to measure blood glucose levels at baseline (0), 30, 60, 90 and 120 min after glucose injection. Blood glucose levels were measured using the blood glucose meter (Onetouch, Johnson & Johnson).

#### Intraperitoneal Insulin Tolerance Test (IPITT)

Mice were fasted 2 h before the day of the IPITT experiment. 1 IU insulin/kg was injected intraperitoneally into experimental mice (Ng et al., 2010; Marmentini et al., 2021). Blood collected from the tail was used to measure blood glucose levels at baseline (0), 30, 60, 90 and 120 min after insulin injection (Onetouch, Johnson & Johnson).

#### Intraperitoneal Pyruvate Tolerance Test (IPPTT)

Mice were fasted overnight (16 h) before IPPTT. On the day of the experiment, the animals received intraperitoneal injection of 2 g pyruvate/kg body weight (Chiang et al., 2014). Blood collected from the tail was used to measure blood glucose levels at baseline (0), 30, 60, 90 and 120 min after pyruvate injection (Onetouch, Johnson & Johnson).

#### Small RNA Library Construction and Sequencing

Mature sperms were isolated from the epididymal tail and vas deferens of F0 father, and RNA was isolated as previously described (Chen et al., 2016). Small RNA library construction and sequencing were performed by BGI (Shenzhen, China). Total RNA was extracted from the mature sperms using Trizol (Invitrogen, Carllsbad, CA, United States). Then it was qualified and quantified using a Nano Drop (NanoDrop, Madison, United States) and Agilent 2100 bioanalyzer (Agilent, Santa Clara, United States). Subsequently, total RNA was purified by electrophoretic separation on a 15% urea denaturing polyacrylamide gel electrophoresis (PAGE) gel and small RNA regions corresponding to the 18–30 nt bands in the marker lane (14–30 ssRNA Ladder Marker, TAKARA) were excised and recovered. Then the small RNAs were ligated to adenylated 3′ adapters annealed to unique molecular identifiers (UMI), followed by the ligation of 5′ adapters. The adapter-ligated small RNAs were subsequently transcribed into cDNA by Superscript II Reverse Transcriptase (Invitrogen, United States) and then several rounds of PCR amplification with PCR Primer Cocktail and PCR Mix were performed to enrich the cDNA fragments. The library was quality and quantitated in two methods: check the distribution of the fragments size using the Agilent 2100 bioanalyzer, and quantify the library using real-time quantitative PCR (QPCR) (TaqMan Probe). The final ligation PCR products were sequenced using the DNBSEQ platform (BGI, Shenzhen, China).

#### Small RNA Data Processing and Analysis

Sequencing reads were processed by using the software SPORTS (Shi et al., 2018). Briefly, clean reads were aligned to mouse reference genome, miRNA datasets, rRNA and YRNA datasets, the genomic tRNA datasets, the mitochondrial tRNA datasets, piRNA datasets, the non-coding RNA datasets by using Bowtie (Langmead et al., 2009) with one mismatch allowance. Differentially expressed tsRNA were detected by using R package edgeR (Robinson et al., 2010), a tsRNA was considered to be significantly differentially expressed when the *p* value is ≤0.05 and the |log2 fold change|≥1. 5′tsRNA-Gly-GCC target gene prediction were performed by Miranda (Enright et al., 2003).

### Western Blot

Mice (14-week-old) were sacrificed by cervical dislocation. Fresh liver tissue or cells were dissolved in lysis buffer which includes 50 mM Tris, 150 mM NaCl, 1 mM EDTA, 1 mM EGTA, 0.1% SDS, 0.5% sodium deoxyholate (D6750, sigma) and 1% Triton X-100. After homogenization, centrifuged at 4°C for 30 min at 16,000 × *g*, supernatant was collected and the protein concentration was determined by the BCA protein assay reagent kit (Pierce). 20–40 μg protein was used for a sodium dodecyl sulfate-polyacrylamide gel electrophoresis (SDS-PAGE) and then electrotransferred to the nitrocellulose membrane (Millipore, Germany). The membrane was blocked with 5% milk in phosphate buffered saline/0.1% Tween 20 (PBST) for 2 h to remove nonspecific binding. After incubation with primary antibody overnight at 4°C, the membrane was washed with 1×PBST and incubated with the horseradish peroxidase-conjugated secondary antibodies at room temperature for 1 h. The membrane blots were detected by the ECL chemiluminescence system as previously described (Wang et al., 2021). The immunoreactive bands were captured on the autoradiographic film, and the density of the bands was analyzed using alpha ease image analysis software (version 3.1.2). Antibodies Sirt6 (sc-517196, 1:1000), FoxO1 (sc-374427, 1:200), Ubiquitin (sc-8017, 1:500, Santa Cruz Biotechnology, DE, CA, United States), Ac-FoxO1 (AF2305, 1:500, Affinity Biosciences, China), PEPCK (16754-1-AP, 1:1000), G6pase (22169-1-AP, 1:1000) (Proteintech, Wuhan, China), and β-actin (A5538, 1:5000, Bimake, Houston, TX, United States) were used. All antibodies were diluted with PBST.

### Nuclear and Cytoplasmic Extraction

Mice (14-week-old) were sacrificed by cervical dislocation. The liver tissue was dissected on ice and homogenized in a glass homogenizer to disrupt the cell membrane and release the cytoplasmic contents. The Nuclear Protein Extraction Kit (P0028, Beyotime Biotechnology, Shanghai, China) was used for nuclear and cytoplasmic extraction. Cell fraction extraction was performed according to the manufacturer’s kit manual.

### Co-Immunoprecipitation (Co-IP)

Mice (14-week-old) were sacrificed by cervical dislocation. The fresh liver tissue was lysed with modified lysis buffer (50 mM Tris, 150 mM NaCl, 1 mM EDTA, 1 mM EGTA, 0.5% sodium deoxyholate and 1% Triton X-100) using 1×protease inhibitor (Bimake, United States). Homogenates were centrifuged at 4°C for 30 min at 13,200 rpm as previously described (Wang et al., 2017). Protein samples were pretreated with 20 μl protein A/G agarose beads (sc-2003, Santa Cruz Biotechnology) for 2 h. After gentle spinning, the supernatant was incubated with FoxO1 antibody at 4°C. The next day, 50 μl protein A/G agarose beads were added and incubated at room temperature for 2 h. The beads were washed three times in ice cold lysis buffer, and then eluted and boiled in 2×loading buffer for Western blotting.

### Real Time Quantitative RT-PCR

Mice (14-week-old) were sacrificed by cervical dislocation. Total RNA was extracted from liver tissues or cells using Trizol (9109, TaKaRa, Japan) according to the instructions. Reverse transcription total RNA (2 μg) was performed using the RevertAid First Strand cDNA Synthesis Kit (01036205, Thermo Scientific) as described previously (Zhang et al., 2018b). Real-time PCR was performed using 10 μl of 2×SYBR Green PCR Master Mix (TaKaRa), cDNA (40 ng), 1 μl of specific primers (10 μM) and water to a final volume of 20 μl by a Bio-Rad icycler iQ PCR machine. β-actin and 18S are used as internal control. Isolation and quantification of tsRNA was carried out according to the manufacturer’s protocol, and U6 was used as the internal control for tsRNA quantification. Each sample was taken in triplicate and the mean was calculated. The relative ratio of RNA expression was calculated with a 2^−ΔΔCt^ method. Primer sequences were listed in Supplementary Table S1.

### tsRNA Mimics/Inhibitor and Transfection

Hepa1-6 cells were cultured in DMEM supplemented with 10% FBS at 37°Cwith 5% CO_2._ The mimics or tsRNA inhibitors were synthesized by Gene Pharma and brought into the Hepa1-6 cells by transient transfection with Lipo 2000 (Thermo Scientific), applying standard conditions to a final concentration of 50 nmol/L. In this study, a random non-targeted mimic tsRNA sequence was introduced as a negative control. The sequence information for 5′tsRNA-Gly-GCC or the negative control mimic and inhibitor are shown below. 5′tsRNA-Gly-GCC mimic: 5′-GCA​UUU​GUG​GUU​CAG​UGG​UAG​AAU​UCU​CGC-3′; NC mimic: 5′-UUG​UAC​UAC​ACA​CAA​AAG​UAC​UG-3′; 5′tsRNA-Gly-GCC inhibitor: 5′-GCG​AGA​AUU​CUA​CCA​CUG​AAC​CAC​AAA​UGC-3′; NC inhibitor: 5′-CAG​UAC​UUU​UGU​GUA​GUA​CAA-3′.

### Luciferase Reporter Gene Assay

Based on bioinformatics predictions, Sirt6 3′-UTR or mutated sequences were subcloned into the pmirGLO Dual-Luciferase vector. To test the interaction between the 3′-UTR of Sirt6 and 5′tsRNA-Gly-GCC, 100 nM 5′tsRNA-Gly-GCC mimic or negative control was co-transfected with 100 ng of wild-type Sirt6 3′-UTR or mutant 3′-UTR into Hepa1-6 cells by Lipofectamine 2000. After 24 h of transfection, luciferase activity was determined according to the manufacturer’s protocol (DL101-01, Vazyme Biotech, Nanjing, China) (Wu et al., 2019). The normalized values (firefly activity/renilla activity) were used for analysis.

### Glucose Production Assay

Glucose production assay was performed as described previously (Dou et al., 2018). After transfection of Hepa1-6 cells with 5′tsRNA-Gly-GCC mimic or inhibitor, the cells were washed five times with PBS and then stimulated with 2 mM sodium pyruvate and 20 mM sodium lactate in glucose-free and serum-free DMEM medium for 18 h. The glucose concentration in the medium was analyzed using a glucose assay kit (GAHK20, Sigma) and normalized to the total protein content determined from whole cell extracts.

### Northern Blots

Northern Blot analysis was carried out as previously described (Zhang et al., 2018a). Isolated sperm RNAs were denatured at 75°C for 3 min and separated in 15% urea-PAGE. The gels were then strained with SYBR GOLD, imaged and immediately transferred to Roche Nylon Membranes (Roche, catalog number: 11417240001) under 100 mA for 35 min, and UV-cross-linked at 0.12 J energy. Membranes were then pre-hybridized with Roche DIG hybridization buffer (Roche, catalog number: 11796895001) for 30 min at 42°C on the shaker with 50 rpm. DIG labelled probe (tRNA-Gly: 5′-DIG-AATTCTACCACTGAACCACCCATGC-3′) was added to the hybridization solution and incubated overnight at 42°C on the shaker with 50 rpm. The membranes were then washed twice (15 min per time) at 42°C with 2 × SSC and 0.1% SDS and twice washes (15 min per time) with 0.1 × SSC and 0.1% SDS at 42°C; and one-time wash at room temperature with 1 × SSC for 10 min. Then, the membrane was blocked by 5% blocking buffer (Roche, catalog number: 11096176001) at room temperature for 3 h. After which, the Anti-Digoxigenin-AP Fab fragments (Roche) was added at a ratio of 1:10000 and incubated for 1 h at room temperature. The membrane was washed in 1 × Maleic acid buffer and 0.3% Tween-20 for 4 × 15 min, and subjected to develop buffer for 10 min and coated with CSPD reagent (Roche, catalog number: 11755633001) at 37°C for 15 min away from light. The image was captured by Tanon 4600 chemiluminescent imaging system (Tanon, Shanghai, China).

### Statistical Analysis

All data were expressed as mean ± SEM. GraphPad Prism 8.0 (GraphPad Software, Inc.) was used for data analyses. Differences between two groups were conformed to a normal distribution before Student’s t test. The differences among groups were compared with two-way analysis. *p* values <0.05 were considered statistically significant.

## Results

### Paternal High-Fat Diet Leads to Gluconeogenesis Alteration in Offspring

**FIGURE 1:**
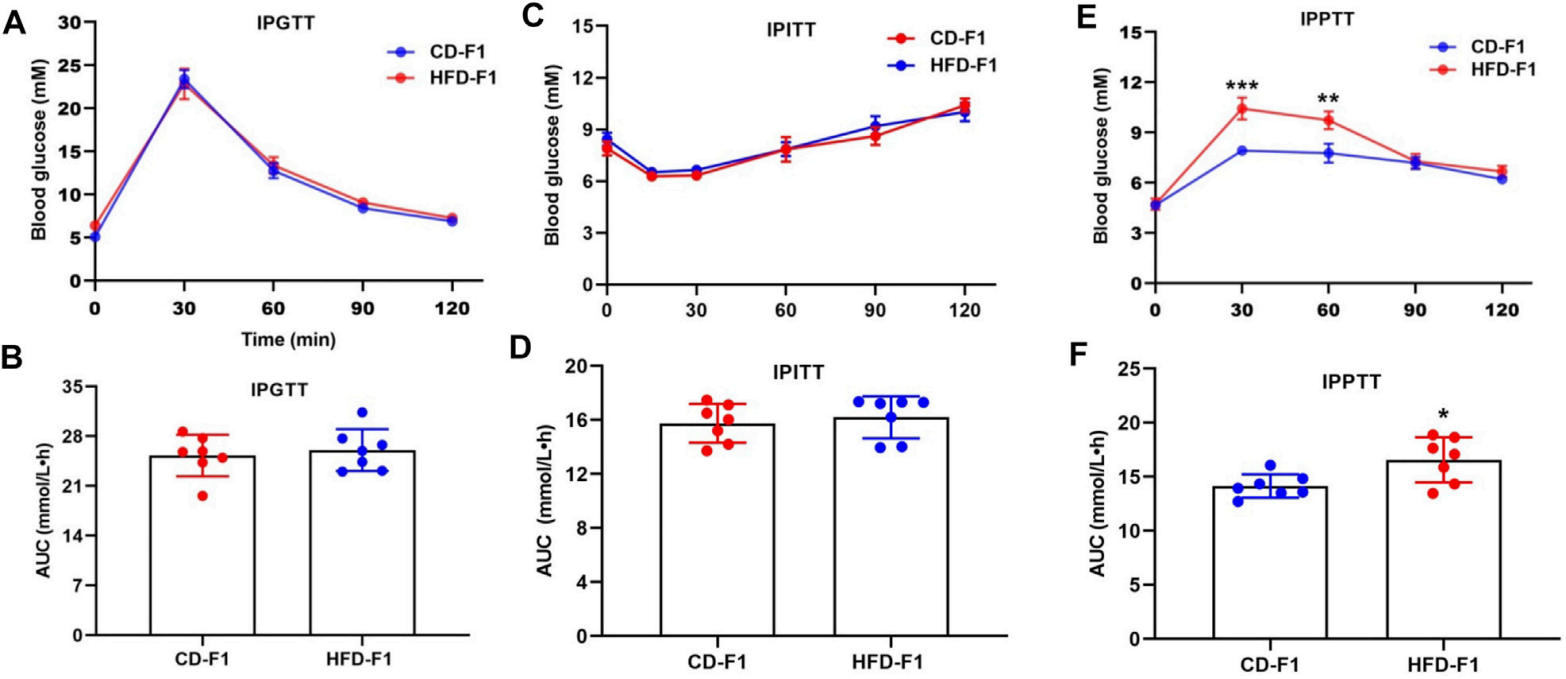
Paternal high-fat diet leads to gluconeogenesis alteration in offspring. **(A**,**B)** Glucose tolerance test (2 g/kg body weight) and AUC of 11-week-old CD-F1 (*n* = 7) and HFD-F1 (*n* = 7) male mice. **(C**,**D)** Insulin tolerance test (1 IU/kg body weight) and AUC of 12-week-old CD-F1 (*n* = 7) and HFD-F1 (*n* = 7) male mice. **(E**,**F)** Pyruvate tolerance test (2 g/kg body weight) and AUC of 13-week-old CD-F1 (*n* = 7) and HFD-F1 (*n* = 7) male mice. *p < 0.05, **p < 0.01, ***p < 0.001.

A growing number of studies have found that paternal high-fat diets are strongly associated with offspring metabolic phenotypes (Wu et al., 2021). We randomly assigned 4-week-old male mice to a control diet or a high-fat diet and found that the weight of HFD-F0 mice increased significantly (Supplementary Figure S1A). Through metabolism related tests, it was found that HFD-F0 mice showed decreased glucose tolerance (Supplementary Figures S2A,B), pyruvate tolerance (Supplementary Figures S2C,D) and insulin sensitivity (Supplementary Figures S2E,F) compared with CD-F0 mice. F0 Males from each group were mated with 8-week-old normal females to produce F1 offspring. Through weight measurement, it was found that there was no significant difference between HFD-F1 group and CD-F1 group (Supplementary Figure S1B). The examination of metabolic phenotype of F1 offspring showed that compared with CD-F1, HFD-F1 male mice were almost unaffected in glucose tolerance (Figures 1A,B) and insulin sensitivity (Figures 1C,D), but the blood glucose level of IPPTT increased (Figures 1E,F), indicating that these offspring had enhanced hepatic gluconeogenesis.

### Paternal High-Fat Diet Leads to Differential Expression of tsRNAs in HFD-F0 Spermatozoa and its Target Genes are Associated With Metabolic Pathways

**FIGURE 2:**
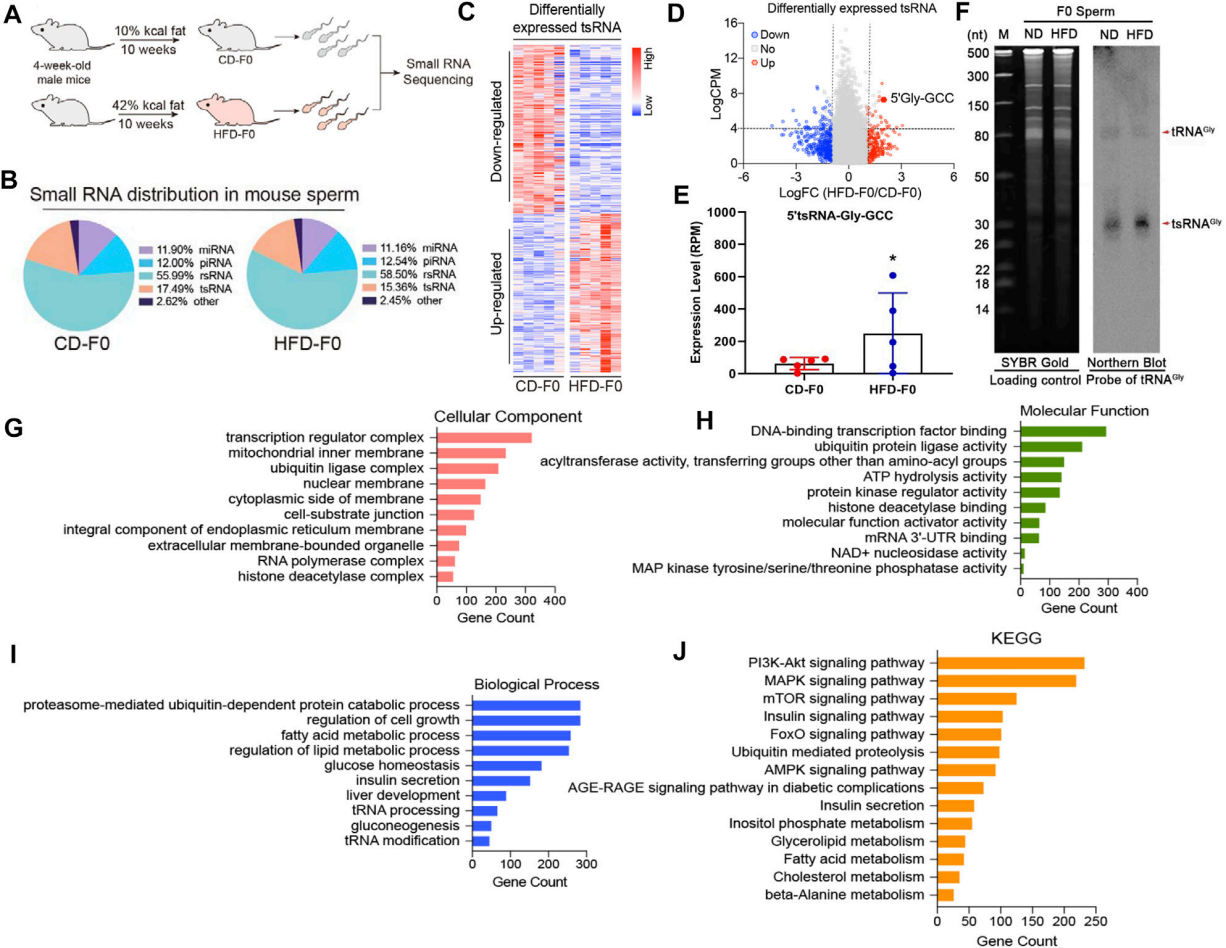
Paternal high-fat diet leads to differential expression of tsRNAs in HFD-F0 spermatozoa and its target genes are associated with metabolic pathways. **(A)** Two groups of 4-week-old male mice were randomly assigned to receive a control diet (CD; 10% kcal fat) or a high-fat diet (HFD; 42% kcal fat) after 10 weeks of feeding, and sperm were isolated for small RNA sequencing. **(B)** Types of small non-coding RNAs identified in spermatozoa from CD-F0 and HFD-F0 subjects. **(C)** Heat map of differential expression tsRNAs across all 10 samples. **(D)**Volcano plot showing the differential expression tsRNA between CD-F0 and HFD-F0 subjects. Red points denote the upregulated tsRNAs and blue points indicate the downregulated ones in HFD-F0 compared to CD-F0 across all 10 samples. **(E)** Expression levels of 5′tsRNA-Gly-GCC (30 nt) in spermatozoa between CD-F0 and HFD-F0 (n = 5). **(F)** Northern blot analyses of 5′tsRNA-Gly (shown by arrow heads) in CD-F0 and HFD-F0, Sperm total RNAs extracted run on a PAGE gel shown as a loading control. **(G**–**I)** Gene Ontology (GO) classification for predicted target genes of 5′tsRNA-Gly-GCC in three categories (cellular component and molecular function and biological process). **(J)** Kyoto Encyclopedia of Genes and Genomes (KEGG) functional enrichment analysis for the predicted target genes of 5′tsRNA-Gly-GCC. *p < 0.05.

Growing evidence suggests that chronic paternal dietary impairment induces the transmission of metabolic phenotypes in offspring mediated by sperm sncRNAs, of which sperm tsRNAs are paternal epigenetic factors that may mediate the intergenerational inheritance of diet-induced metabolic disorders (Chen et al., 2016; Sarker et al., 2019). We then performed small RNA sequencing to obtain a profile of small RNA in spermatozoa from the CD-F0 and HFD-F0 groups (Figure 2A) and a comparative analysis of differentially expressed RNA in spermatozoa from both groups with |Log2Fold Change|>1 and *p*< 0.05 were set as filtering conditions (Figure 2B). The heat map generated by hierarchical cluster analysis shows similar spectral clusters and samples in each group (Figure 2C). Among the small RNA biotypes including miRNAs, piRNAs, rsRNAs and tsRNAs, sperm tsRNAs showed sensitive alteration under high fat diet conditions. In the differentially expressed tsRNAs, 329 were up-regulated and 319 were down regulated in the HFD-F0 group compared with the CD-F0 group (Figure 2D), including tRNA-Gly-GCC 5′ end derived small RNAs (25 nt–32 nt) - 5′tsRNA-Gly-GCC (Figure 2E), which was significantly increased in HFD-F0 group. Northern blot results have confirmed the upregulation of 5′tsRNA-Gly-GCC in sperm of HFD F0 mice (Figure 2F). To further understand the potential function of 5′tsRNA-Gly-GCC in regulating the metabolic alteration phenotype of the HFD offspring, we applied Miranda to identify the potential target mRNAs for 5′tsRNA-Gly-GCC between HFD-F0 and CD-F0 groups. The GO analysis on cellular component, molecular function and biological process all indicated that 5′tsRNA-Gly-GCC was mainly involved in ubiquitin-dependent protein catabolic process and participated in regulation of cell growth, fatty acid metabolic process and insulin secretion (Figures 2G–I). KEGG analysis showed that the predicted target genes were mainly involved in PI3K-Akt, MAPK, mTOR, Insulin, FoxO, AMPK signaling pathway and metabolism (Figure 2J), which may be related to excessive activation of liver gluconeogenesis in HFD-F1 mice.

### Altered Sirt6-FoxO1 Signaling Associated With Hepatic Gluconeogenesis in HFD-F1 Mice

**FIGURE 3:**
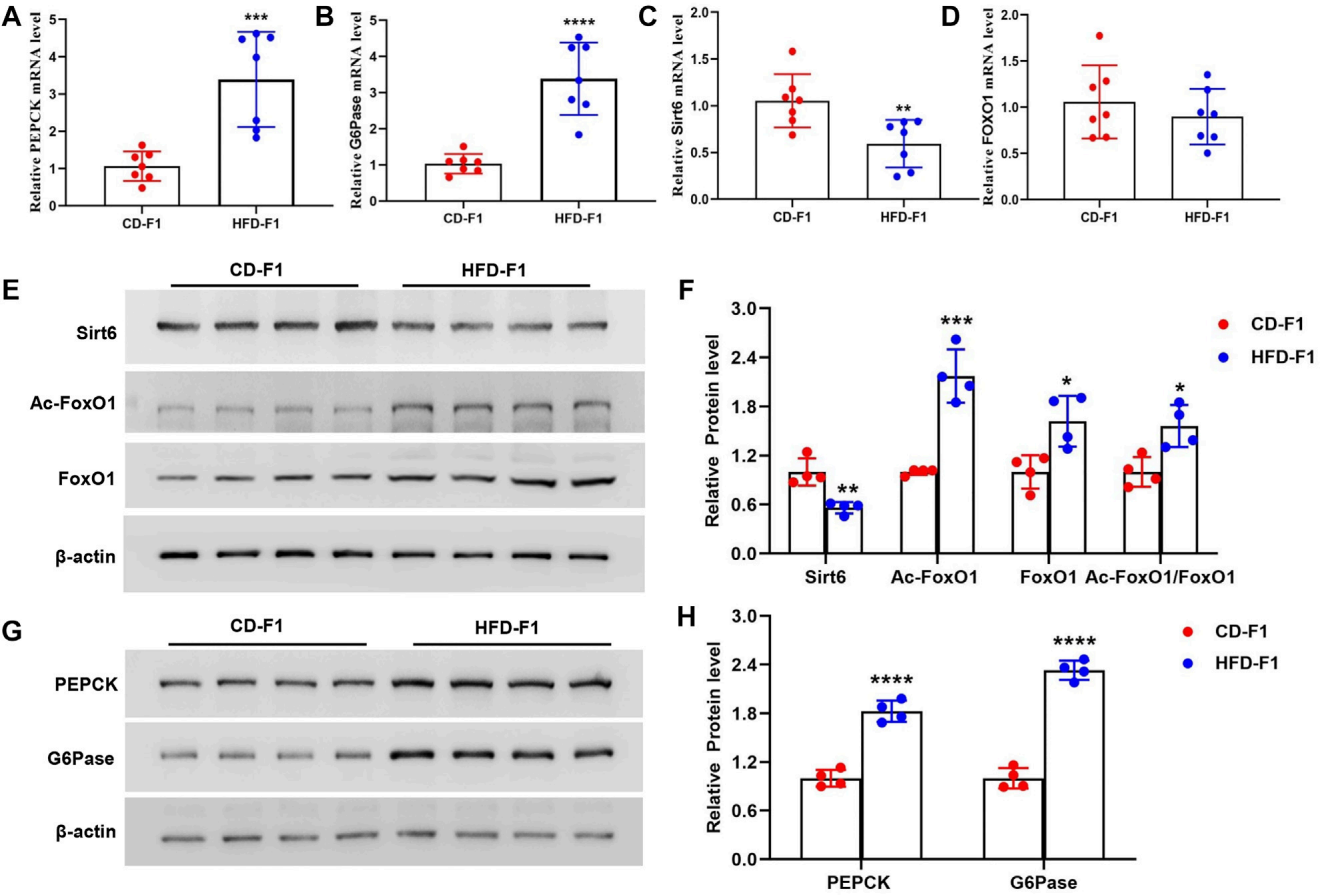
Altered Sirt6-FoxO1 signaling associated with hepatic gluconeogenesis in HFD-F1 mice. **(A**–**D)** PEPCK **(A)**, G6Pase **(B)**, Sirt6 **(C)** and FoxO1 **(D)** mRNA level in liver tissue from HFD-F1 mice and CD-F1 mice was examined by Real-time PCR (*n* = 7). **(E**,**F)** Sirt6, Ac-FoxO1, FoxO1 and Ac-FoxO1/FoxO1 protein expression in liver tissue from HFD-F1 mice and CD-F1 mice was detected by western blot analysis (*n* = 4). **(G**,**H)** PEPCK and G6Pase protein expression in liver tissue from HFD-F1 mice and CD-F1 mice was detected by western blot analysis (*n* = 4). *p < 0.05, **p < 0.01, ***p < 0.001, ****p < 0.0001.

Since HFD-F1 mice exhibit enhanced hepatic gluconeogenesis, to explore the mechanism of gluconeogenesis dysfunction, we examined the expression levels of PEPCK and G6Pase, two key enzymes involved in the regulation of gluconeogenesis. Compared with CD-F1 mice, the levels of PEPCK and G6Pase mRNA in liver tissue of HFD-F1 mice were significantly increased (Figures 3A,B). Sirtuins (Sirt) are highly conserved NAD^+^-dependent protein deacetylases with seven members, of which Sirt6 is abundantly expressed in the liver. Sirt6 is interacted with forkhead box protein O1 (FoxO1), a key transcription factor that activates PEPCK and G6Pase and inhibits hepatic gluconeogenesis, and leads to FoxO1 deacetylation (Schilling et al., 2006; Zhang et al., 2014). Compared with CD-F1 mice, Sirt6 mRNA level was significantly lower in liver tissue of HFD-F1 mice, but the mRNA level of FoxO1 was unchanged (Figures 3C,D). Compared with CD-F1 group, Sirt6 protein in liver tissue of HFD-F1 mice decreased, while Ac-FoxO1, FoxO1 and Ac-FoxO1/FoxO1 were significantly up-regulated (Figures 3E,F). In addition, PEPCK and G6Pase in liver tissue of HFD-F1 mice also increased dominantly (Figures 3G,H). Therefore, the altered Sirt6-FoxO1 signaling in HFD-F1 mice liver might be associated with the inherited abnormal hepatic gluconeogenesis from HFD-F0 mice.

### 5′tsRNA-Gly-GCC Promotes Gluconeogenesis in Hepa1-6 Cells

**FIGURE 4:**
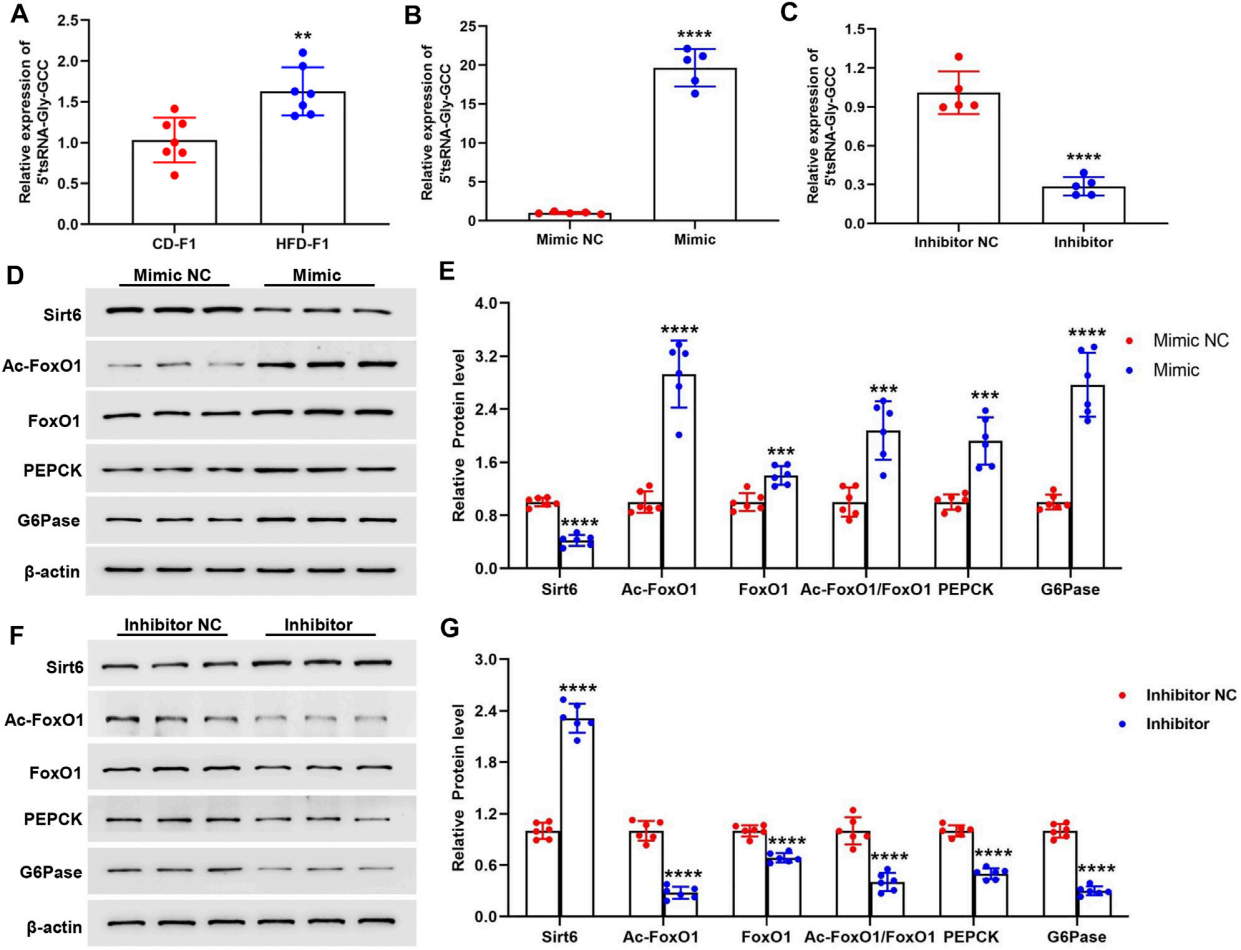
5′tsRNA-Gly-GCC promotes gluconeogenesis in Hepa1-6 cells. **(A)** 5′tsRNA-Gly-GCC level in liver tissue from HFD-F1 mice and CD-F1 mice was examined by Real-time PCR (*n* = 7). **(B**,**C)** Real-time PCR analysis of 5′tsRNA-Gly-GCC expression level after 5′tsRNA-Gly-GCC mimic **(B)** and inhibitor **(C)** or non-targeted RNA (NC) into hepa1-6 cells 24 h (*n* = 5). **(D**,**E)** Hepa1-6 cells transfected with 5′tsRNA-Gly-GCC mimic 48 h, Sirt6, Ac-FoxO1, FoxO1, Ac-FoxO1/FoxO1, PEPCK and G6Pase protein expression was detected by western blot analysis (*n* = 6). **(F**,**G)** Hepa1-6 cells transfected with 5′tsRNA-Gly-GCC inhibitor 48 h, Sirt6, Ac-FoxO1, FoxO1, Ac-FoxO1/FoxO1, PEPCK and G6Pase protein expression was detected by western blot analysis (*n* = 6). **p < 0.01, ***p < 0.001, ****p < 0.0001.

Our previous data showed that the sperm enriched tsRNAs were mainly derived from the 5′ end of mature tRNAs with nucleotide length ranging from 29 nt to 34 nt, and were sensitively changed both in expression profiles and RNA modifications levels under high-fat diet conditions (60% fat), which could act as epigenetic information carrier and transmit certain metabolic traits from father to offspring (Chen et al., 2016). Here in a 42% high-fat diet mice model, we identified that the sperm 5′tsRNA-Gly-GCC was significantly increased in HFD-F0 mice (Figure 2C). Interestingly, we found that the expression levels of 5′tsRNA-Gly-GCC in liver tissue of HFD-F1 mice was also significantly increased compared with CD-F1 mice (Figure 4A), suggesting that there might be a regulation correlation between 5′tsRNA-Gly-GCC in HFD-F0 mice sperm and HFD-F1 mice liver. To further clarify the biological function of upregulated 5′tsRNA-Gly-GCC in offspring metabolic alteration on gluconeogenesis, we transfected synthetic 5′tsRNA-Gly-GCC mimic and inhibitor into Hepa1-6 cells. Successful 5′tsRNA-Gly-GCC overexpression and knockdown were detected by Real-time PCR (Figures 4B,C). Hepa1-6 cells transfected with 5′tsRNA-Gly-GCC mimic significantly inhibited Sirt6 expression, promoted the expression of Ac-FoxO1, FoxO1 and Ac-FoxO1/FoxO1, and led to an increase in PEPCK and G6Pase expression (Figures 4D,E). In addition, transfected with 5′tsRNA-Gly-GCC mimic, the gluconeogenesis level was increased in Hepa1-6 cells (Supplementary Figure S3A). Consistent with this, transfection of Hepa1-6 cells with 5′tsRNA-Gly-GCC inhibitor significantly promoted Sirt6 expression, inhibited the expression of Ac-FoxO1, FoxO1, Ac-FoxO1/FoxO1, PEPCK, G6pase (Figures 4F,G) and reduced the gluconeogenesis level (Supplementary Figure S3B). Therefore, our data showed that 5′tsRNA-Gly-GCC could promote gluconeogenesis in cells, associated with the regulation of Sirt6-FoxO1 signaling pathway.

### 5′tsRNA-Gly-GCC Targets 3′UTR of Sirt6 mRNA

**FIGURE 5:**
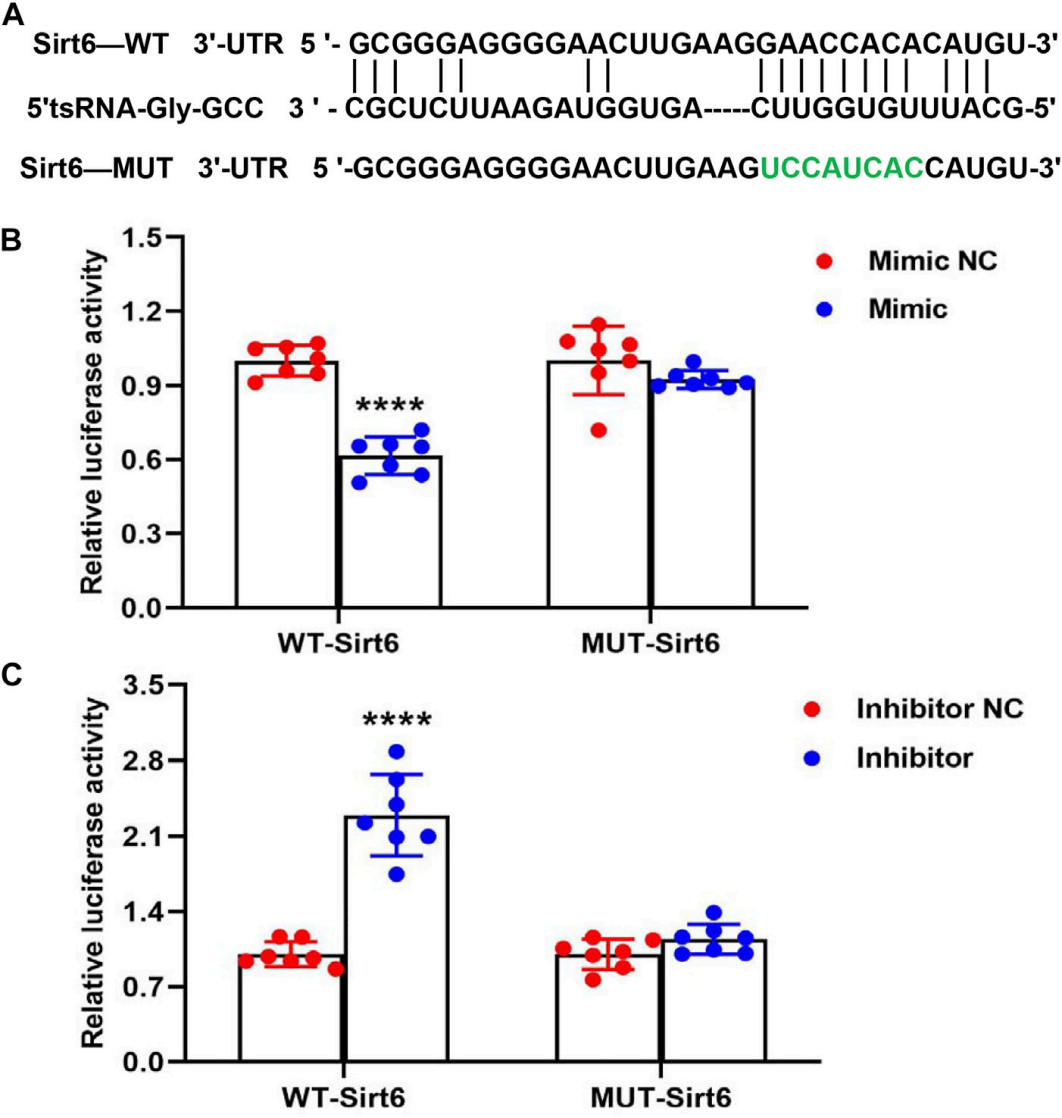
5′tsRNA-Gly-GCC targets 3′UTR of Sirt6 mRNA. **(A)** An illustration of the putative site of 5′tsRNA-Gly-GCC binding within the 3′UTR of Sirt6 predicted by bioinformatics software RNAhybrid, with green used to highlight those nucleotides mutated for a mutant version of this sequence. **(B)** Luciferase activity assay revealed that 5′tsRNA-Gly-GCC mimic transfection into Hepa1-6 cells resulted in a 40% reduction in luciferase activity for constructs containing a WT but not a mutated version of this 3′UTR of Sirt6 binding site (*n* = 7). **(C)** Luciferase activity assay revealed that 5′tsRNA-Gly-GCC inhibitor transfection into Hepa1-6 cells significantly increased WT Sirt6 3′UTR activity, while 5′tsRNA-Gly-GCC inhibitor also had no significant effect on MUT Sirt6 3′UTR (*n* = 7). ****p < 0.0001.

In order to further study the potential target of 5′tsRNA-Gly-GCC in Sirt6-FoxO1 signaling pathway, we then predicted the regulation target of 5′tsRNA-Gly-GCC by the bioinformatics software RNAhybrid. The data showed that 5′tsRNA-Gly-GCC might regulate the expression of Sirt6 in a base-paired dependent manner (Figure 5A). According to the predicted target binding site, the WT and MUT plasmids of Sirt6 3′UTR were constructed to validate the direct interaction effectiveness between tsRNA-Gly-GCC and Sirt6. We co-transfected the plasmid with 5′tsRNA-Gly-GCC mimic or inhibitor into Hepa1-6 cells, and the results of the luciferase reporter gene assay showed that the 5′tsRNA-Gly-GCC mimic reduced WT Sirt6 3′UTR activity, but had no significant effect on the MUT Sirt6 3′UTR (Figure 5B). Consistent with this, 5′tsRNA-Gly-GCC inhibitor elevated WT Sirt6 3′UTR activity, while had no significant effect on MUT Sirt6 3′UTR (Figure 5C). These results suggest that 5′tsRNA-Gly-GCC can interact with Sirt6 3′UTR, and thus have a targeting effect.

### FoxO1 Activation Caused by Reduced FoxO1 Nuclear Exclusion and Ubiquitination Degradation in HFD-F1 Liver

**FIGURE 6:**
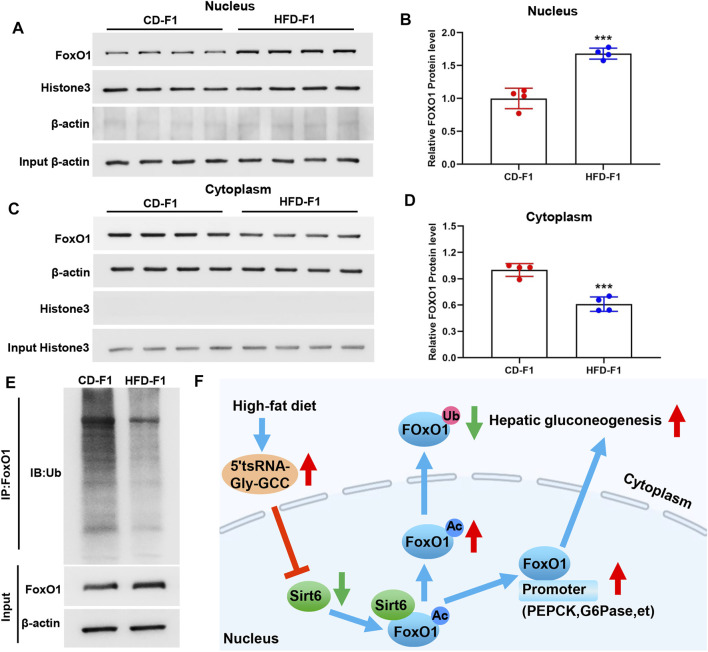
FoxO1 activation caused by reduced FoxO1 nuclear exclusion and ubiquitination degradation in HFD-F1 liver. **(A**–**D)** Cytoplasmic and nuclear FoxO1 in liver tissue from HFD-F1 mice and CD-F1 mice was examined by Western blotting. Cytoplasmic protein β-actin and nuclear protein histone3 served as controls. (*n* = 4). **(E)** Liver tissue from HFD-F1 mice and CD-F1 mice were collected for the immunoprecipitation with FoxO1 antibody. FoxO1 and ubiquitinated FoxO1 protein were examined by Western blotting. **(F)** Schematic diagram of the mechanism by which 5′tsRNA-Gly-GCC regulates the Sirt6-FoxO1 pathway involved in enhanced gluconeogenesis in the offspring as a result of the paternal high-fat diet. ***p < 0.001.

Recent studies have shown that deacetylation of FoxO1 is a prerequisite for its nuclear translocation, and Sirt6 specifically interacts with FoxO1 to promote its deacetylation and nuclear exclusion (Zhang et al., 2014). The nuclear exclusion of FoxO1 would promote the degradation of its ubiquitin-proteasome system (Yamagata et al., 2008). In addition, studies have shown that high-fat diet feeding leads to a significant increase in the nuclear content of FoxO1 protein (Li et al., 2009). In the liver tissue of HFD-F1 mice, FoxO1 increased in the nucleus (Figures 6A,B) and decreased in the cytoplasm (Figures 6C,D), which was consistent with the decrease of Sirt6 of HFD-F1. In addition, Co-IP experiments revealed a significant reduction in ubiquitinated degradation of FoxO1 in the liver tissue of HFD-F1 mice, which was consistent with the reduction of nuclear exclusion of FoxO1 (Figure 6E). Therefore, the paternal high-fat diet induced upregulation of sperm 5′tsRNA-Gly-GCC might be responsible for the increased 5′tsRNA-Gly-GCC levels in HFD-F1 liver, which would inhibit the expression levels of Sirt6 in the liver tissue of HFD-F1 mice, leading to the increase of acetylation degree of FoxO1, the decrease of FoxO1 nuclear exclusion and its ubiquitination degradation. As a result, FoxO1 was activated, the expression of key gluconeogenesis genes PEPCK and G6Pase increased, and gluconeogenesis was enhanced in the liver of HFD-f1 mice (Figure 6F).

## Discussion

Epigenetic alteration induced by environmental exposure could reprogram the health and behavior of offspring. Paternal dietary components affect the metabolic system of offspring through epigenetic markers transmitted between generations (Ng et al., 2010; de Castro Barbosa et al., 2016b; Castro Barbosa et al., 2019), however, the underlying mechanism is still not clear. There is increasing evidence that long-term paternal dietary pattern, such as low protein diet and HFD exposure, can induce the transmission of sperm sncRNAs mediated metabolic phenotype in offspring (Sharma et al., 2016; Zhang et al., 2018a; Sarker et al., 2019). Among the different subpopulations of sperm RNA, it is reported that tsRNAs constitute the main part of sncRNAs. Although the biological function of tsRNA is unclear, the potential link between sperm tsRNAs levels and obesity has been observed in human and animal studies (Chen et al., 2016). A recent human study has shown that more tsRNAs are present in the sperm of obese men compared to healthy lean individuals (Donkin et al., 2016). In HFD mice, we previously found that a subset of tsRNAs, mainly from the 5′ transfer RNA half showed altered expression profiles and RNA modifications. Microinjection of purified sperm tsRNA components from HFD males into normal zygotes causes metabolic disorders in F1 offspring and alters metabolic gene expression in both early embryos and islets of F1 offspring. However, chemical synthesizing tsRNAs that highly expressed in HFD sperm and injecting them into normal offspring did not induce an HFD metabolic phenotype in the F1 offspring. It is possible that sperm tsRNAs contain various RNA modifications that could affect the stability of RNA (Chen et al., 2016). These evidences give a strong support that the RNA modifications in sperm tsRNAs indeed have pivotal roles in the paternal intergenerational epigenetic inheritance. However, in this study we only synthesized the 5′tsRNA-Gly-GCC that had been found increased in F0 sperm for the experiment to study whether the 5′tsRNA-Gly-GCC could target Sirt6 and induced the metabolic disorder in hepal-6 cells, and we did not test whether the synthesized tsRNAs without modification could transfer the paternal acquired phenotype to the offspring. Therefore, although sperm tsRNAs could represent a paternal epigenetic factor, which may mediate the intergenerational inheritance of diet induced metabolic alteration, the mechanisms of how tsRNAs regulating intergenerational inheritance still need to be further investigated.

In this study, we provide evidence that a paternal high-fat diet (42% fat) reprograms the epigenome of mice sperm. We demonstrated that a high-fat diet upregulated 5′tsRNA-Gly-GCC expression in the sperm of F0, and this is consistent with previous reports that low protein diet resulted in an approximately 2-3 fold increase of tRNA-Gly-GCC in 5′ fragment in sperm (Sharma et al., 2016). In addition, the increased expression of 5′tsRNA-Gly-GCC was also found in the liver tissues of HFD-F1 mice (Figure 4A). Sirtuins are highly conserved NAD^+^-dependent protein deacetylases, and among the seven members of the family, Sirt6 is considered to show greater hope in prolonging life than other sirtuins (Kanfi et al., 2012). Recently, it has been reported that Sirt6 plays a central role in regulating DNA damage response and glucose metabolism (McCord et al., 2009; Kaidi et al., 2010; Mao et al., 2011; Dominy et al., 2012). FoxO1, a key transcription factor that activates phosphoenolpyruvate carboxykinase (PEPCK) and glucose-6-phosphatase (G6Pase), plays an important role in promoting gluconeogenesis (Zhang et al., 2014). Sirt6 can specifically interact with FoxO1 to promote its deacetylation and nuclear exclusion, resulting in loss of FoxO1-induced gluconeogenesis (Zhang et al., 2014). Our study found that liver tissue of HFD-F1 mice showed a decrease of Sirt6, a significant increase in Ac-FoxO1, FoxO1, PEPCK and G6Pase, and a decrease in FoxO1 nuclear exclusion and degradation. We also synthesized 5′tsRNA-Gly-GCC mimic and inhibitor and transfected Hepa1-6 cells to further confirm the regulation of Sirt6 by 5′tsRNA-Gly-GCC and changes in FoxO1 signal and gluconeogenesis. TsRNAs showed multifaceted function in cellular translational regulation, which include but not limited to replacing the translational initiator factors, binding with ribosome RNA and proteins that inhibited the entrance of mRNA in ribosome, base-paring with ribosome mRNA and impede translation (Gebetsberger et al., 2017; Shi et al., 2019). Interestingly, the tsRNAs targeting sites are located not only in the 3′-UTR but also in the 5′-UTR and CDS regions of target mRNAs (Shi et al., 2019), suggesting a base-pair depended mechanism of tsRNAs in regulating the expression levels of their targets. In this study, prediction by RNAhybrid software revealed that 5′tsRNA-Gly-GCC can directly regulate the Sirt6 3′UTR. By constructing WT and MUT plasmids of Sirt6 3′UTR and co-transfecting them with 5′tsRNA-Gly-GCC mimic and inhibitor, we further confirmed that 5′tsRNA-Gly-GCC was indeed capable to regulate the expression levels of Sirt6 in a base-depended anti-sense-like manner.

Our findings support that alteration in tsRNAs causes metabolic phenotypes in offspring. So far, it is not clear how the alterations in sperm tsRNAs lead to changes in tsRNAs in other tissues of the offspring. Sarker et al. showed microinjection of sperm tsRNAs from F1-HFD males into normal zygotes reproduced the obesogenic phenotype and addiction-like behavior and caused alterations in genes which were related to neuronal growth, differentiation, axonal guidance, and dendritic spine formation and maturation in the brain, but the role of tsRNAs in the brain is also unclear (Sarker et al., 2019). It is worth pondering whether this metabolic phenotype caused by high paternal high-fat induced tsRNAs alteration in our study can be transmitted across generations. There is increasing evidence that sperm small noncoding RNAs (sncRNAs) play an important role in regulating DNA methylation, histone modification and mRNA transcription (Pauli et al., 2011; Sarker et al., 2019). Mature sperm contain multiple sncRNAs, such as miRNA, piRNA, and tsRNA. Sperm RNA studies in intergenerational and intergenerational epigenetic contexts have shifted the focus to determining the role of specific subgroups of sncRNAs (Casas and Vavouri, 2014; Sarker et al., 2019). In addition, our previous study found that DNMT2 (DNA methyltransferase 2) deletion prevented the elevation of RNA modifications (m^5^C, m^2^G) in the 30–40 nt RNA fraction of sperm induced by HFD. Also, Dnmt2 deletion altered the expression profile of sperm small RNAs, including levels of tsRNAs and rRNA-derived small RNAs (rsRNA-28S) and eliminated the transmission of high-fat diet (HFD)-induced metabolic disorders to offspring (Zhang et al., 2018a). However, the intervention methods need to be further improved and studied. Therefore, to investigate the relationship between altered sperm tsRNAs caused by paternal high-fat diet and abnormal metabolism in offspring is still our future research direction. In addition, the effects of high-fat diet induced alterations in tsRNAs modifications are worthy of further exploration.

In summary, the present study demonstrated that 5′tsRNA-Gly-GCC was abnormally upregulated in the sperm of HFD-F0 mice and in the liver tissues of HFD-F1 mice, which can further regulate Sirt6-FoxO1 pathway and promote gluconeogenesis in HFD-F1 mice liver, indicating that 5′tsRNA-Gly might be one of the functional parts that programmed the F1 metabolic alteration during the intergenerational epigenetic inheritance of paternal acquired traits.

## Data Availability

The raw data used for this study has been deposited into Gene Expression Omnibus with the accession number GSE196881 (https://www.ncbi.nlm.nih.gov/geo/query/acc.cgi?acc=GSE196881).
