# Supplementary figures and images for: Paternal High-Fat Diet Altered Sperm 5'tsRNA-Gly-GCC Is Associated With Enhanced Gluconeogenesis in the Offspring
Source: Front Mol Biosci. 2022 Apr 11;9:857875. doi: 10.3389/fmolb.2022.857875 (PMC9035875; doi:10.3389/fmolb.2022.857875)

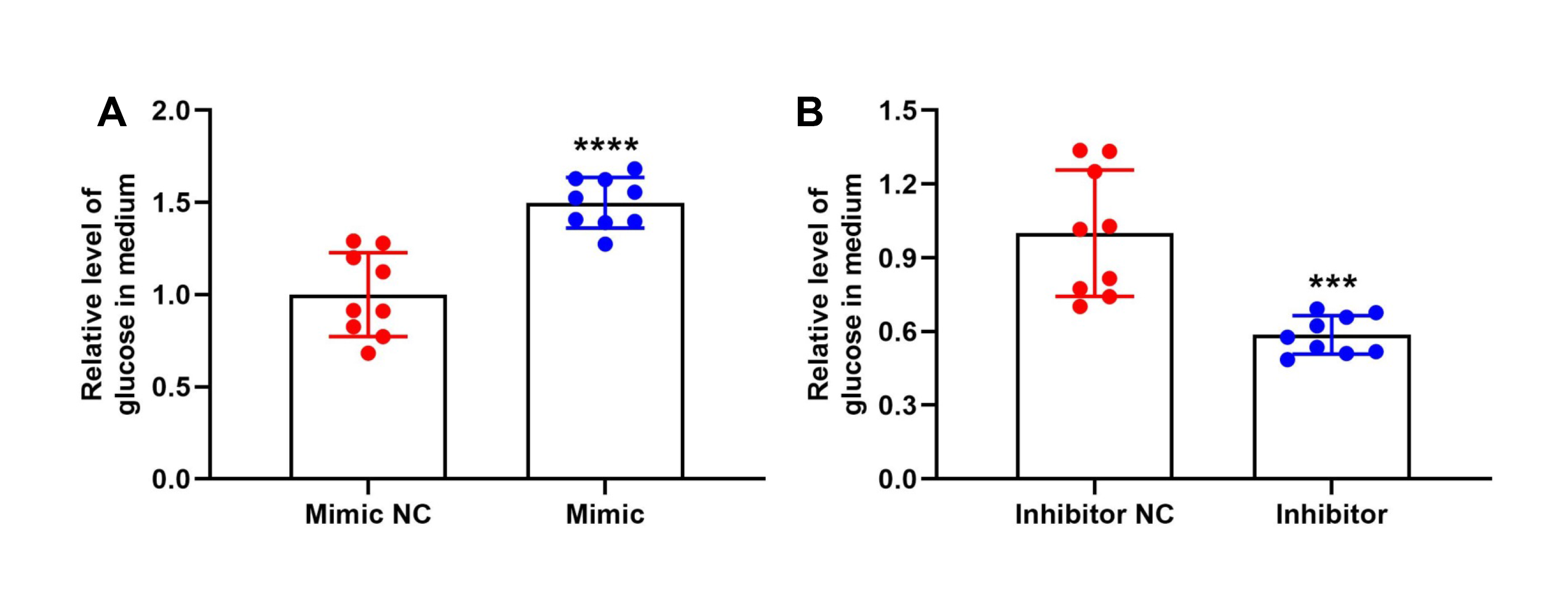

Supplement: Supplementary file 1 [file Image3.TIF]

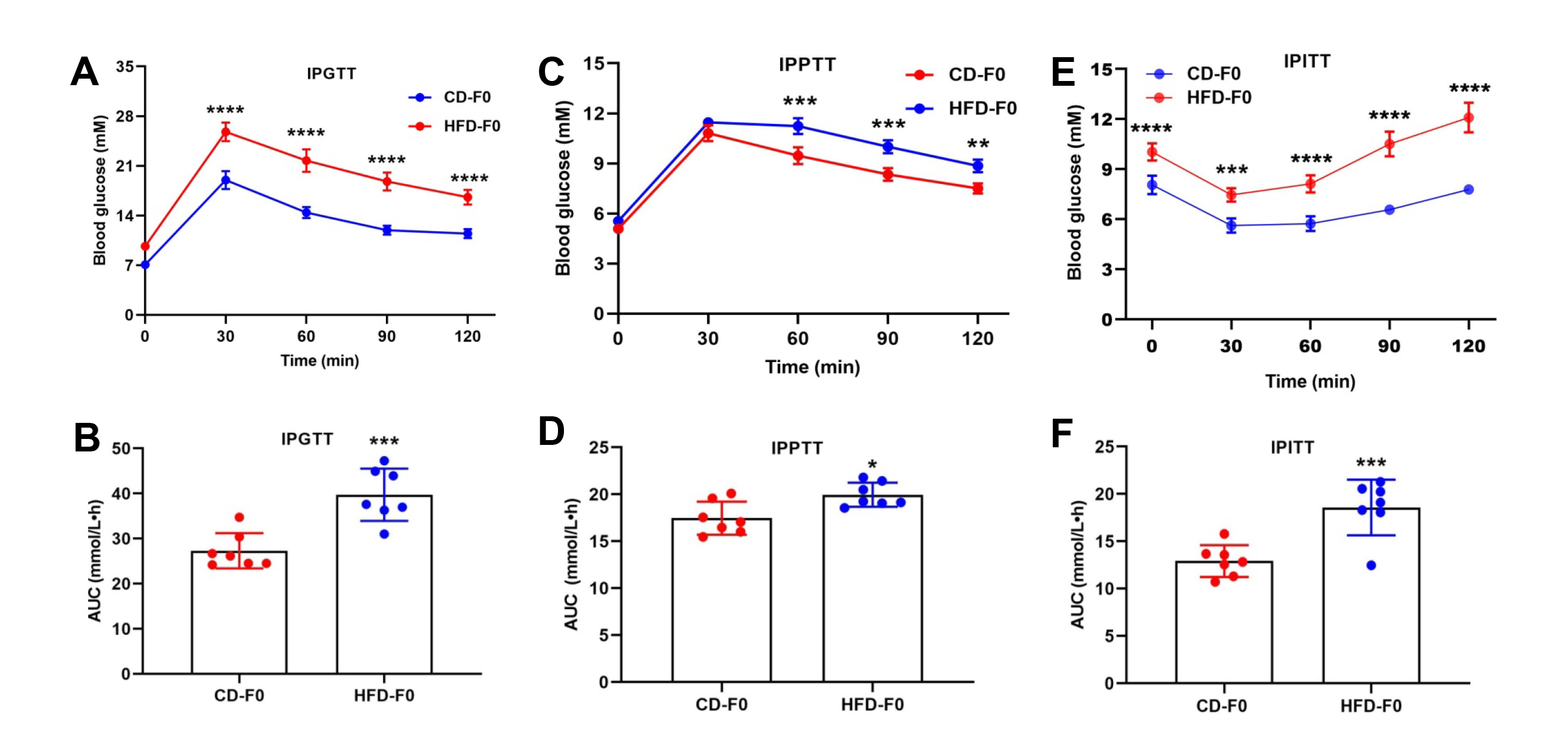

Supplement: Supplementary file 2 [file Image2.TIF]

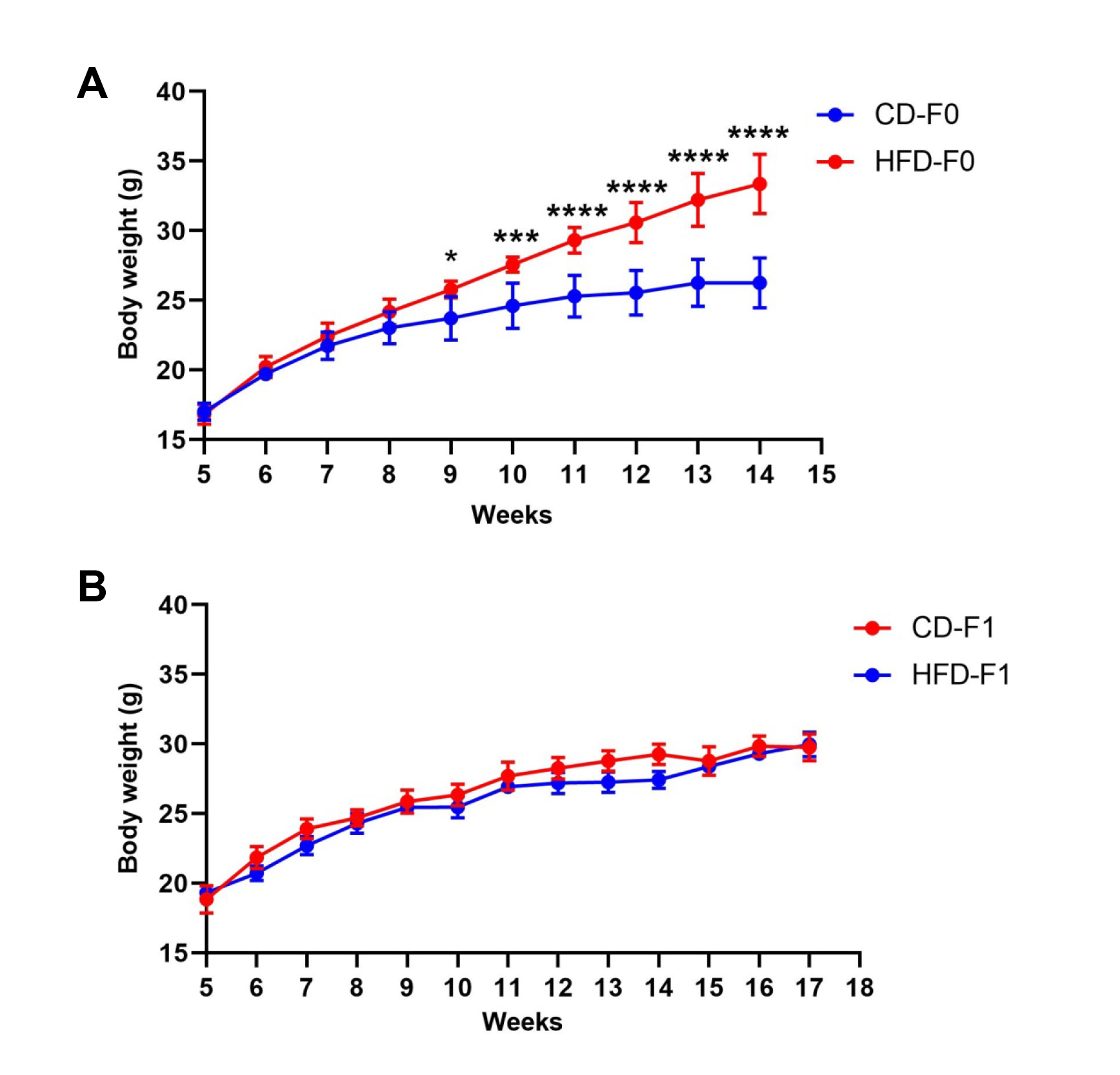

Supplement: Supplementary file 3 [file Image1.TIF]
